# Attenuation of Hippocampal Evoked Potentials *in vivo* by Activation of GtACR2, an Optogenetic Chloride Channel

**DOI:** 10.3389/fnins.2021.653844

**Published:** 2021-03-29

**Authors:** Anirudh R. Acharya, Lars Emil Larsen, Wouter Van Lysebettens, Wytse Jan Wadman, Jean Delbeke, Kristl Vonck, Alfred Meurs, Paul Boon, Robrecht Raedt

**Affiliations:** 4BRAIN Team, Department of Head and Skin, Faculty of Medicine and Health Sciences, Ghent University, Ghent, Belgium

**Keywords:** hippocampus, CA1 inhibition, GtACR2, chloride channel, optogenetics, evoked potential

## Abstract

**Aim:**

GtACR2, a light-activated chloride channel, is an attractive tool for neural inhibition as it can shunt membrane depolarizations. In this study, we assessed the effect of activating GtACR2 on *in vivo* hippocampal CA1 activity evoked by Schaffer collateral (SC) stimulation.

**Methods:**

Adult male Wistar rats were unilaterally injected with 0.5 μL of adeno associated viral vector for induction of GtACR2-mCherry (*n* = 10, GtACR2 group) or mCherry (*n* = 4, Sham group) expression in CA1 pyramidal neurons of the hippocampus. Three weeks later, evoked potentials (EPs) were recorded from the CA1 subfield placing an optrode (bipolar recording electrode attached to an optic fiber) at the injection site and a stimulation electrode targeting SCs. Effects of illumination parameters required to activate GtACR2 such as light power densities (LPDs), illumination delays, and light-pulse durations were tested on CA1 EP parameters [population spike (PS) amplitude and field excitatory postsynaptic potential (fEPSP) slope].

**Results:**

In the GtACR2 group, delivery of a 10 ms light-pulse induced a negative deflection in the local field potential which increased with increasing LPD. When combined with electrical stimulation of the SCs, light-induced activation of GtACR2 had potent inhibitory effects on CA1 EPs. An LPD of 160 mW/mm^2^ was sufficient to obtain maximal inhibition CA1 EPs. To quantify the duration of the inhibitory effect, a 10 ms light-pulse of 160 mW/mm^2^ was delivered at increasing delays before the CA1 EPs. Inhibition of EPs was found to last up to 9 ms after the cessation of the light-pulse. Increasing light-pulse durations beyond 10 ms did not result in larger inhibitory effects.

**Conclusion:**

Precisely timed activation of GtACR2 potently blocks evoked activity of CA1 neurons. The strength of inhibition depends on LPD, lasts up to 9 ms after a light-pulse of 10 ms, and is independent of the duration of the light-pulse given.

## Introduction

Optogenetics refers to a technique, where neurons are genetically modified to express non-endogenous light-sensitive proteins (opsins) to reversibly modulate their activity with a high degree of spatiotemporal precision using a specific wavelength of light. Light-induced activation of cation-conducting opsins such as ChR2 is generally used to excite neurons, while anion-conducting opsins such as NpHR are used to inhibit neurons (Zhang et al., 2007). While optogenetic tools for excitatory opsins are most frequently used, inhibitory opsins are desirable to study the function of specific brain regions and brain networks (Wietek et al., 2015). Many *in vivo* studies using inhibitory opsins have resorted to inward chloride pumps (e.g., halorhodopsins; NpHR) (Sadler et al., 2017; Lee et al., 2018; Moon et al., 2018) or outward proton pumps (e.g., archaerhodopsins; ArCh) (Falgairolle and O’Donovan, 2019; Hoseini et al., 2019; Solecki et al., 2019). These opsins, however, have certain limitations. First, both are pumps that may disrupt neuronal physiology (e.g., excessive chloride load or extreme pH values) if stimulation paradigms are not carefully considered (Raimondo et al., 2012). Second, they have slower conductance compared to channels because each opsin can transfer only one ion per photochemical cycle. In practice, efficient inhibition of neurons with these opsins requires high levels of opsin expression on the cell membrane and require higher light powers (Wietek et al., 2015). Photostimulation sensitivity of these opsins, measured as effective power density for 50% activation (EPD50), is low (NpHR and ARCh, EPD50: 5–10 mW/mm^2^) (Mattis et al., 2011). So, applications of these opsins are further limited to brief inhibitions of neurons (typically < 15 s), as prolonged illumination with higher light powers causes heating of tissue which is an unwanted side-effect (Wiegert et al., 2017).

Contrary to opsin pumps, GtACR2 is a natural blue-light activated anion-channel rhodopsins (ACRs), found in the algae *Guillardia theta* (Govorunova et al., 2015). It can inhibit neurons by shunting the membrane potential to the equilibrium potential of chloride. Since it is a channel and not a pump, non-physiological changes in the chloride gradient are prevented. Compared to other inhibitory opsins like NpHR and even engineered ACRs like ChloC, GtACR2 utilizes lower light powers for activation (EPD50: 0.05 mW/mm^2^) and is highly selective for chloride anions, while it can still generate large currents (Govorunova et al., 2015). These features make GtACR2 a valuable research tool for neuronal inhibition. However, its effect on population-level neurotransmission and neuronal excitability is unknown.

The layered structure of the hippocampus makes it relatively straightforward to measure *in vivo* evoked potentials (EPs) using extracellular recording electrodes. The CA1 EPs induced by Schaffer collateral (SC) stimulation is a well-characterized electrophysiological recording to evaluate excitability of neuronal circuitry. Activation of the SC generates a field excitatory postsynaptic potential (fEPSP) which appears as a negative potential in the stratum radiatum (i.e., active sink) and a positive potential caused by a passive return current in the stratum pyramidale/oriens (i.e., passive source) of CA1 subfield. When SC stimulation is sufficient to trigger action potentials in CA1 neurons, a population spike (PS) emerges as a sharp negative potential in stratum pyramidale/oriens (i.e., active sink) (Graziane and Dong, 2016). Further, paired electrical pulses can be used to evaluate pre- or postsynaptic mechanisms of short-term plasticity, such as paired-pulse facilitation (PPF) and paired-pulse depression (PPD). The change in the paired pulse relations of EP parameters indicates a change in the probability of neurotransmitter release, as well as a change of postsynaptic excitability which could be due to various mechanisms such as change in postsynaptic receptor affinity, density and expression, ionic conductance, and signal transduction pathways (Regehr, 2012; Jackman and Regehr, 2017). So, we used CA1 EPs to evaluate the *in vivo* effects of light-induced activation of GtACR2 on population-level excitability of neurons.

## Materials and Methods

### Animals

Adult male Wistar rats (8 weeks old, 300–400 g, *n* = 14) from Janvier (Hannover, Germany) were used. The animals were housed in a temperature and humidity regulated room with a 12 h light/dark cycle (lights “on” between 8 AM and 8 PM). Water and food were provided *ad libitum*. All procedures were approved by the Animal Experimental Ethical Committee of Ghent University (ECD16/31). Treatment and care were complied with the ARRIVE guidelines.

### Intrahippocampal Viral Vector Injection

The rats were anesthetized with isoflurane (5% in medical O_2_ for induction and 2% for maintenance). Body temperature was maintained at 35 ± 1°C using a heating pad with a rectal probe for temperature feedback. The rats were then placed in a stereotactic frame; a single incision was made on the shaved heads to expose the skull. After clearing the periosteum, a burr-hole was made using an electric micro-drill tool. Ten rats were unilaterally injected with 0.5 μL of AAV2/7-CamKIIα-0.4-intron-hGtACR2-mCherry (1.8 × 10^12^ genome copies/mL) (Leuven Viral Vector Core, Belgium) using a Hamilton syringe (Model 7001, Hamilton Co., United States, 0.1 μL/min flow rate) in CA1 [-5.0 mm anteroposterior, +3.0 mm mediolateral to bregma, -2.8 mm dorsoventral to brain surface (GtACR2 group)]. Four rats were unilaterally injected with 0.5 μL of AAV2/7-CamKIIα-0.4-intron-mCherry (6.0 × 10^12^ genome copies/mL) at the same coordinates (Sham group). The presence of the CamKIIα promoter in the viral construct ensured expression in excitatory neurons. The mCherry fluorophore was used as a reporter protein. After the injection, the needle was left in place for 5 min to minimize backflow of the viral vector solution due to retraction of the injection needle. Postoperatively, 1 mg/kg Meloxicam was administered subcutaneously for analgesia.

### Experimental Setup

All subsequent data were obtained during a single recording session under isoflurane anesthesia 3 weeks after viral vector injection. Upon exposure of the skull, an epidural screw electrode (1.25 mm diameter; Invivo1) was placed in the left frontal bone and used as ground/reference electrode. Using stereotaxic procedures, a recording optrode (bipolar electrode + optic fiber) was placed at the previously injected site (AP: -5.0 mm, ML: + 3.0 mm, DV: -2.1 to -2.6 mm), and a stimulation electrode was placed in the stratum radiatum of the CA1 (AP: -3.0 mm, ML: +1.5 mm, DV: -2.0 to -2.5 mm). The optrode was custom-made by gluing a multi-mode optic fiber 100 μm away (200 μm diameter, 0.39 NA, Thorlabs, Germany) from the dorsal contact of the bipolar recording electrode (two twisted polyimide coated stainless steel wires of 70 μm diameter, 225 μm tip separation, California Fine Wire, Grover Beach, CA, United States). A quadripolar stimulation electrode (four twisted polyimide-coated stainless steel wires of 120 μm diameter, 300 μm tip separation) with 600 μm tip distance was used for stimulation (California Fine Wire, Grover Beach, CA, United States). The placement of the optrode was guided by the audio of spontaneous firing of neurons. The depths of the optrode and stimulation electrode were adjusted using electrophysiological feedback. The optrode position was optimized until the ventral contact of the electrode was in stratum radiatum (fEPSP represented as negative voltage), and the dorsal contact was positioned in the stratum pyramidale/oriens (fEPSP represented as positive voltage superimposed with a negative PS). Input–output (IO) relationships were determined by applying electrical pulses with increasing current intensity (100–1000 μA in steps of 100 μA) and an interval of 10 s between stimuli. This was repeated four times. For each rat, the lowest stimulation intensity resulting in a maximum amplitude PS (I_max_) (640 ± 65 μA) was selected for all subsequent experimental paradigms.

### Experimental Paradigms

**FIGURE 1:**
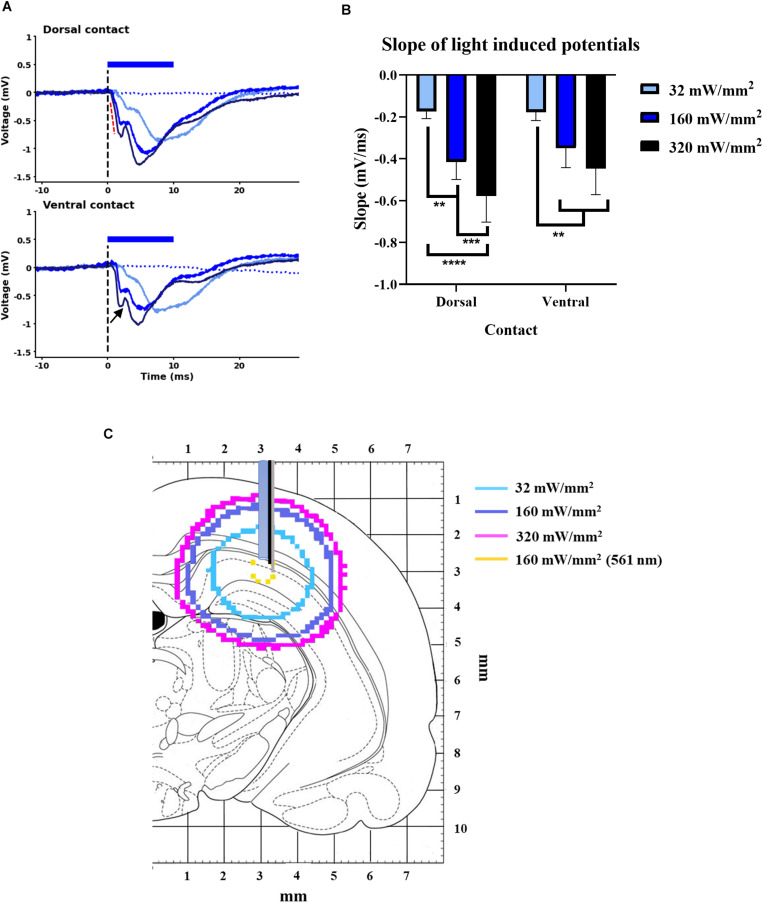
Light-induced potentials from GtACR2 expressing CA1 subfield. **(A)** A negative field potential with compound morphology (black arrowhead) at dorsal and ventral contacts of optrode for the three light-power densities. Black dotted line indicates time of light delivery; in Sham group, no light-induced potential was detected (blue dotted line, 160 mW/mm^2^). **(B)** Slope of light-induced potentials animals (indicated by red dotted line in **A**) increased with higher LPD at both dorsal and ventral contacts of the optrode (*n* = 8). **p < 0.005, ***p < 0.0005, and ****p < 0.0001. **(C)** Fluence contours of 0.05 mW/mm^2^ (i.e., EPD50 of GtACR2) for three light-power densities of blue-light (470 nm) and 5.4 mW/mm^2^ (i.e., EPD50 of eNpHR3.0) for LPD of 160 mW/mm^2^ of yellow-light (561 nm) (Modified from Paxinos and Watson, 1998).

In Experiment 1, we investigated whether illumination alone evoked field potentials in the GtACR2 group (Figure 1A). Light-pulses of 10 ms were delivered at three light power densities (LPDs): 32, 160, and 320 mW/mm^2^. For each LPD, five light-pulses were delivered with an interval of 10 s.

**FIGURE 2:**
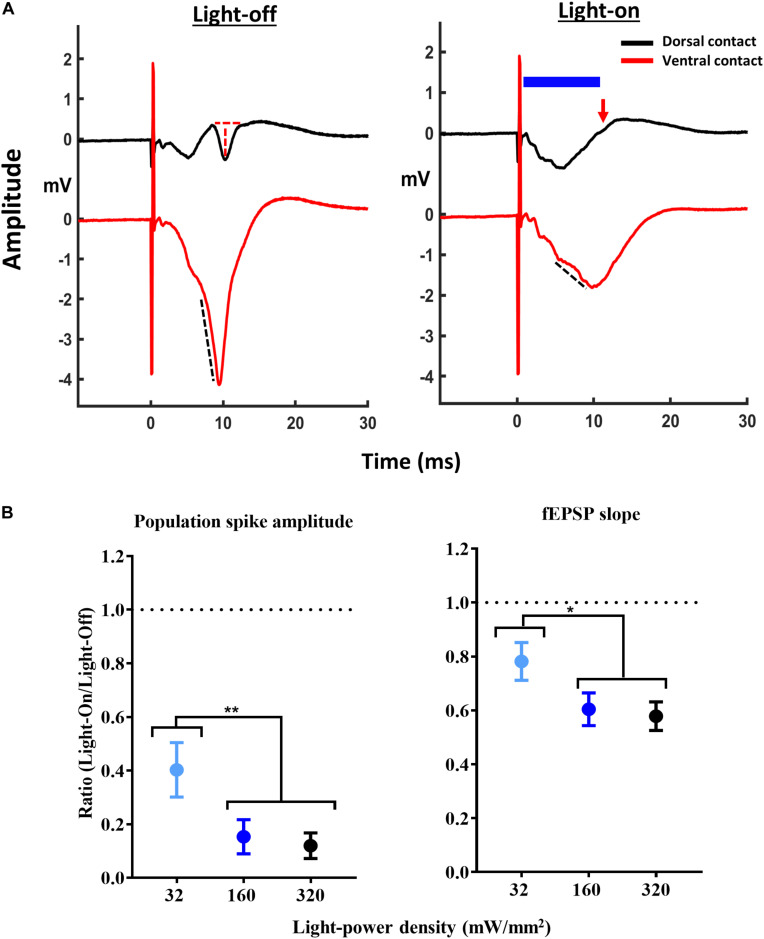
Attenuation of CA1 EPs with activation of GtACR2. **(A)** Representative traces of CA1 EPs without (light-off) and with (light-on) delivery of 10 ms light-pulse (blue bar) at 160 mW/mm^2^, 1 ms after the electrical stimulation at dorsal contact measuring population spike (PS) amplitude (red dotted line and red arrow indicating complete suppression of PS) and ventral contact measuring fEPSP slope (black dotted line). **(B)** Increasing the light-power density resulted in a decrease of the PS amplitude (*n* = 10) and fEPSP slope (*n* = 8). *p ≤ 0.05 and **p < 0.005.

The remaining experiments evaluated the effects of activating GtACR2 on CA1 EPs. In Experiment 2, a CA1 EP was evoked every 10 s at I_max_, while administering a 10 ms light-pulse 1 ms after every other electrical stimulation (Figure 2A). In the GtACR2 group, the effects of 32, 160, and 320 mW/mm^2^ were evaluated. In the Sham group, the effects of 160 mW/mm^2^ were evaluated as this intensity was sufficient to reliably modulate the CA1 EPs of GtACR2 group (i.e., this experiment was performed after retrieving the data of the GtACR2 group). LPD of 160 mW/mm^2^ was used for all subsequent experiments.

**FIGURE 3:**
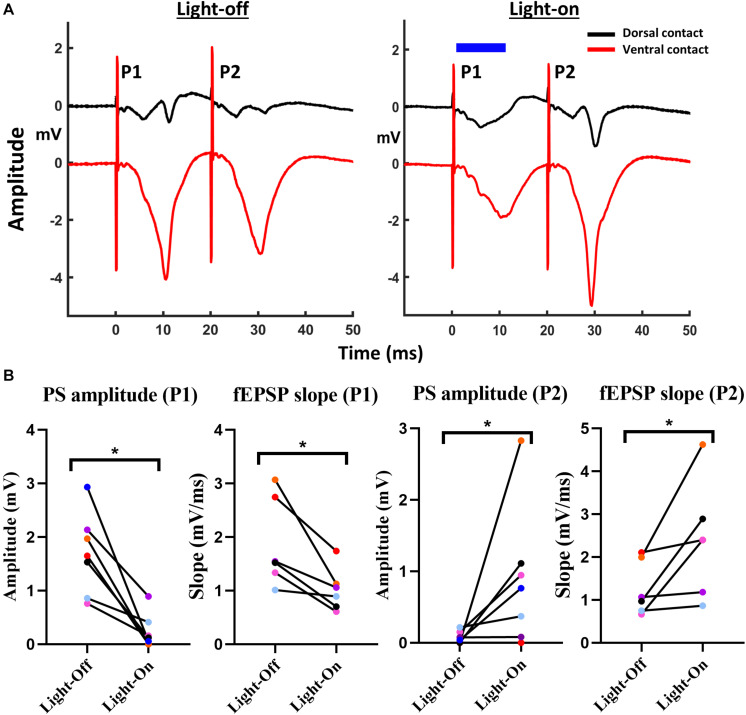
Activation of GtACR2 affected paired-pulse relations of CA1 EPs. **(A)** Representative traces of paired-pulse CA1 EPs without (red trace) and with (blue trace) delivery of 10 ms light-pulse (blue bar) at 160 mW/mm^2^, 1 ms after the electrical stimulation. **(B)** Activation of GtACR2 at P1 resulted in the transition from PPD to PPF as indicated by PS amplitude (*n* = 7) and fEPSP slope (*n* = 6). The graphs represent values of EP parameters for each individual rat for P1 and P2 obtained in light-on versus light-off conditions. *p ≤ 0.05.

In Experiment 3, we investigated the effect of activating GtACR2 on paired-pulse relations of EPs, for which, paired pulses with an inter-pulse interval (IPI) of 20 ms. Initially, the paired pulses were delivered every 10 s without illumination, after which the protocol was repeated while illuminating the first pulse (P1) of the paired stimuli for 10 ms starting 1 ms after electrical stimulation (Figure 3A). For both conditions, five iterations were recorded.

**FIGURE 4:**
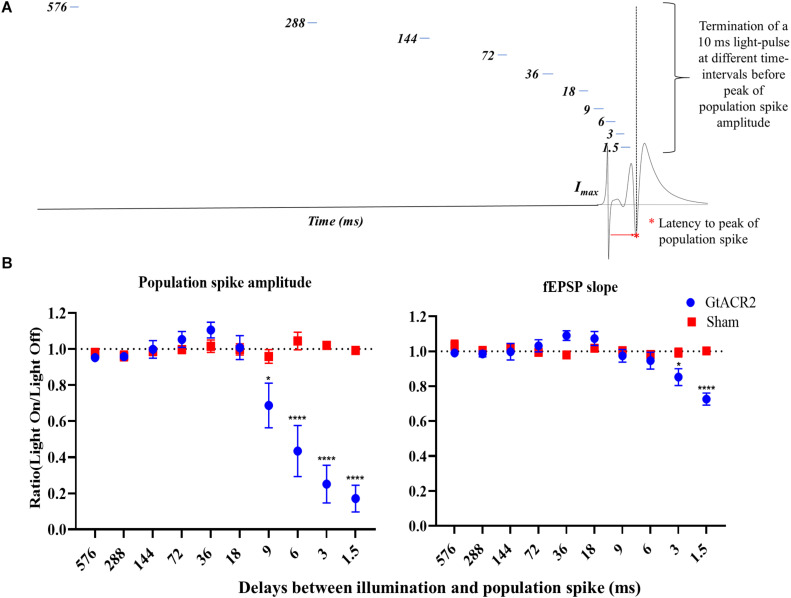
Effect of delays between illumination and CA1 EPs. **(A)** Schematic of the experimental paradigm. **(B)** The inhibitory effect of activating GtACR2 on population spike (*n* = 5) and fEPSP slope (*n* = 4) of CA1 EPs decreased when increasing the delay between a 10 ms light-pulse and PS peak. Mean of the ratios (light-on/light-off) and standard error of the mean (SEM) for the EP parameters are plotted. *p ≤ 0.05 and ****p < 0.0001.

In Experiment 4, we estimated how long the effects of GtACR2 activation outlast a 10 ms light-pulse (Figure 4A). Every minute, two sets of paired EPs (IPI 20 ms) were evoked with 9 s between them. A 10 ms light-pulse was delivered before the second set of paired EPs with an increasing delay between the end of the light-pulse and the first PS (i.e., 1.5, 3, 6, 9, 18, 36, 72, 144, 288, and 576 ms). Three iterations of each delay were recorded.

**FIGURE 5:**
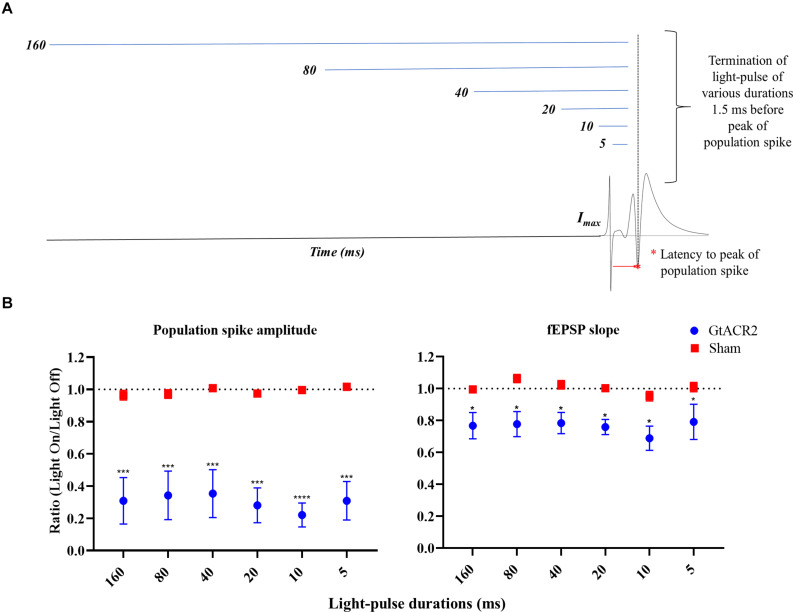
Effect of light-pulse durations on CA1 EPs. **(A)** Schematic of the experimental paradigm. **(B)** The GtACR2-mediated inhibition of EPs was maintained at increasing the light-pulse durations; there was no significant increase of the effect on population spike (*n* = 6) and fEPSP slope (*n* = 5). Mean of the EP light-on/light-off condition parameters’ ratios and the corresponding standard error (SEM) are plotted against for light-pulse durations. A significant difference between the groups was found. *p ≤ 0.05, ***p < 0.0005, and ****p < 0.0001.

In Experiment 5, different illumination durations were evaluated for their effects on CA1 EPs (Figure 5A). Again, every minute we evoked two sets of paired EPs (IPI 20 ms) with 9 s between them. Light-pulses with increasing durations (i.e., 5, 10, 20, 40, 80, and 160 ms) were administered 1.5 ms before the second set of paired EPs. All light-pulses ended 1.5 ms before the first PS. Three iterations of each light-pulse duration were recorded.

### Instrumentation for Electrophysiology and Optogenetics

Analog signals were high-pass filtered at 0.1 Hz, amplified 248 times, and digitized at a 16-bit resolution (±10 V input range) using a USB-6259 NI-DAQ card (National Instruments, Austin, TX, United States). The NI-DAQ card was connected to a computer which was used to control the stimulation and data acquisition using custom-made software. CA1 EPs were sampled at 10 kHz. A TTL-controlled laser (SLOC Lasers, China) was used to deliver blue light (473 nm) through the optic fiber of the optrode. Prior to implantation, laser output was adjusted to achieve the light-power density (LPD) levels 32, 160, and 320 mW/mm^2^ at the optic fiber tip. This was achieved using a handheld light power-meter (PM100A + S120VC, ThorLabs, Germany). A constant-current linear stimulus isolator (Digitimer, United Kingdom) generated charge-balanced square-wave pulses (200 μs per phase) needed to obtain the EPs.

### Histology

At the end of the experiments, rats were deeply anesthetized with an overdose of Thiobarbital (180 mg/kg i.p.) and transcardially perfused with ice-cold phosphate-buffered saline (PBS) followed by paraformaldehyde in 0.1 M phosphate buffer solution (4%, pH 7.4). The brains were isolated and post-fixed overnight in 4% paraformaldehyde. After cryoprotection in 30% sucrose, brains were snap−frozen in 2-methylbutane cooled by liquid nitrogen. Coronal sections of 40 μm thickness were collected using a cryo-microtome (Leica, Germany) set at -20°C. The sections were washed twice with PBS and kept for 30 and 60 min in 0.5 and 1% H_2_O_2_. Later, they were permeabilized and blocked in 0.2% Triton−X−100/0.4% fish skin Gelatin (blocking buffer), after which they were transferred into the primary antibody solution (rabbit anti−RFP, 1:1000, Rockland 600−401−379) and kept overnight at 4°C. On the next day, the sections were washed twice in blocking buffer followed by 1 h in a secondary antibody (goat anti−rabbit Alexafluor 594, 1:1000, Abcam ab150088). After rinsing twice with PBS, 4’,6-diamidino-2-phenylindole (DAPI) was added for nuclear staining (1 μg/mL, Sigma–Aldrich). Slices were mounted on glass slides, Vectashield^®^ anti-fade mounting medium (Vector Laboratories, United States) was applied, and a coverslip was placed. Images were taken using a fluorescence microscope (Carl Zeiss, AxioCAM HR).

### Data Analysis

Data were recorded and analyzed using MATLAB software (MathWorks, United States). The initial slopes of the light-induced potentials, and CA1 EP parameters (PS amplitude and fEPSP slope) at I_max_ were measured. The relative changes of EP parameters due to illumination were assessed by calculating ratios for light-on versus light-off conditions for each individual animal. In Experiment 3, fEPSP slope and PS amplitude for first pulse (P1) and second pulse (P2) were compared between light-on and light-off conditions. All statistical analyses and graphs were prepared using the Prism 8 software (Graphpad, United States). Group means with the standard error of the mean (SEM) were plotted. The data were tested for normal distribution using Shapiro–Wilk’s test. Repeated measures two-way ANOVA and Tukey’s HSD *post hoc* tests were used to compare initial slopes of light-induced potentials for different LPDs in the GtACR2 group. The effects of different LPDs on CA1 EPs were analyzed using repeated measures ANOVA and Tukey’s HSD *post hoc* tests. In the Sham group, a two-tailed one-sample *t*-test was done for LPD of 160 mW/mm^2^. Paired-pulse relations were analyzed using the non-parametric two-tailed Wilcoxon matched-pair test as correlation coefficient for effective pairing was found to be negative. The effects of illumination delay and light-pulse durations on CA1 EPs in the GtACR2 and Sham group were compared only for P1 of paired EPs using repeated measures two-way ANOVA and Geisser-Greenhouse correction for sphericity. Two-tailed *t*-tests with Holm–Šídák correction for multiple comparisons were used to compare different delays and light-pulse durations between the two groups. Significant effects for the results were inferred if *p* ≤ 0.05.

## Results

### Experiment 1: Light-Induced Potentials From GtACR2 Expressing CA1 Subfield

Illuminating the GtACR2-expressing CA1 subfield of the hippocampus with 32, 160, and 320 mW/mm^2^ resulted in negative field potentials at both the dorsal and ventral electrode contacts, positioned near the axons and dendrites of the CA1 neurons, respectively (Figure 1A). The (initial) slope of these potentials increased with higher LPDs at both recording contacts (*n* = 8, *F* = 8.72, *p* < 0.005) (Figure 1B) and did not significantly differ between the contacts. The potentials evoked at ventral contact for an LPD of 160 mW/mm^2^ had about five times smaller amplitude (-0.98 ± 0.41 mV) and slope (-0.35 ± 0.09 mV/ms) compared to fEPSP amplitude (-5.08 ± 0.58 mV) and slope (-1.49 ± 0.21 mV/ms) of electrically induced CA1 EPs. These light-induced potentials were absent in the Sham group. An increase in the slope of these potentials was likely to be associated with an increase in the volume of tissue that is optogenetically modulated. To illustrate this, we estimated the volume contours of tissue where light powers are predicted to exceed the EPD50 of GtACR2 (i.e., 0.05 mW/mm^2^) using a MATLAB script for Monte Carlo simulation of light-distribution in rat brain (Supplementary Method 1 and Figure 1C). For illustrative purposes, we also depicted the volume contour of that could be optogenetically inhibited tissue with an LPD of 160 mW/mm^2^ when the chloride pump eNpHR3.0 is used (EPD50 of 5.4 mW/mm^2^) (Mattis et al., 2011). If assuming the width of lateral light scattering is quantitatively similar to the depth of light scattering from the fiber tip (Yizhar et al., 2011), GtACR2 demonstrated a 67-fold increase in the targeted spherical volume of neural tissue compared to eNpHR3.0.

### Experiment 2: Effect of Light-Power Densities on GtACR2 Driven Modulation of CA1 EPs

In GtACR2 expressing rats 10 ms of illumination attenuated PS amplitude (*F* = 15.68, *p* < 0.005) and fEPSP slope (*F* = 12.39, *p* < 0.005) (Figures 2A,B). For all EP parameters, *post hoc* comparison demonstrated less potent inhibition for 32 mW/mm^2^ compared to 160 mW/mm^2^ (*p* < 0.05) and 320 mW/mm^2^ (*p* < 0.05). Since 160 and 320 mW/mm^2^ resulted in a similar suppression of the CA1 EP, we decided to use the 160 mW/mm^2^ for subsequent experiments. In the Sham group, 160 mW/mm^2^ had no effect on the CA1 EPs (*n* = 4) (Supplementary Figure 1).

### Experiment 3: Effect of GtACR2 Activation on Paired-Pulse Relations

Optogenetic inhibition of P1 (PS amplitude: *W* = −28, *p* < 0.05, and fEPSP slope: *W* = −21, *p* < 0.05) in the paired pulse protocol resulted in an increased P2 response (Figure 3A). Both the PS amplitude (*W* = 21, *p* < 0.05) as the fEPSP slope (*W* = 21, *p* < 0.05) increased significantly for P2 under illumination conditions (Figure 3B).

### Experiment 4: Effect of Delays Between Illumination and CA1 EPs

To assess how long the inhibitory effect of GtACR2 activation remains after cessation of illumination, a 10 ms light-pulse with increasing delays between the end of the light-pulse and the PS peak was administered (Figure 4A). An interaction between the groups and delay was found for PS amplitude (*F* = 34.10, *p* < 0.0001) and fEPSP slope (*F* = 9.75, *p* < 0.0001) (Figure 4B). Only in the GtACR2 group, an effect of delay was seen on PS amplitude (*F* = 30.88, *p* < 0.0001) and fEPSP slope (*F* = 10.18, *p* < 0.0005). The results indicated a more potent inhibition for the shortest delays. *Post hoc* comparison between the Sham and GtACR2 groups demonstrated that 10 ms light-pulses significantly reduced PS amplitude when the delay between light-pulse and PS was less or equal to 9 ms, while fEPSP slope was only reduced for delays up to 3 ms (*p* < 0.05).

### Experiment 5: Effect of Light-Pulse Durations on CA1 EPs

We also tested whether, besides timing, the inhibition of CA1 EPs varied with the light-pulse duration (Figure 5A). All tested light-pulse durations ended 1.5 ms before the PS peak which was shown to be the most efficacious for CA1 EP suppression in Experiment 3. No significant interaction between the groups and light-pulse durations could be identified (*p* > 0.05). A significant difference between the groups was found for PS amplitude (*F* = 20.56, *p* < 0.005) and fEPSP slope (*F* = 10.38, *p* < 0.05) (Figure 5B). *Post hoc* comparison between the Sham and GtACR2 groups showed attenuation of all EP parameters in the GtACR2 group for all the light-pulse durations (*p* < 0.05).

### Histological Verification of GtACR2-mCherry Expression

**FIGURE 6:**
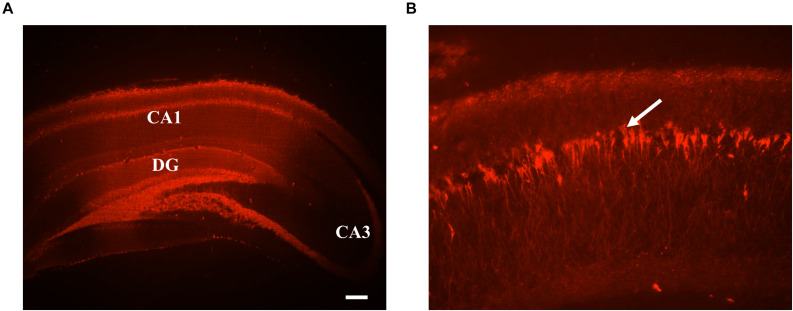
**(A)** Representative histological images of coronal sections of rat brain hippocampus expressing GtACR2-mCherry at the site of recording. GtACR2 expressing neurons appear in red color. The scale bar measures 200 μm. **(B)** Magnified view of a small area of CA1, GtACR2-mCherry neurons designated by the arrow.

Epifluorescence microscopy 3 weeks after viral vector injection confirmed mCherry expression in excitatory CA1 neurons due to the presence of CamKIIα promoter in the gene construct (Liu and Jones, 1996). Widespread expression could be observed in all layers of hippocampal CA1 region (stratum radiatum, pyramidale, and oriens), CA3, and dentate gyrus (Figures 6A,B). As responses recorded were local evoked activity at CA1-subfield, activation of GtACR2 at dentate gyrus is inconsequential.

## Discussion

In this study, we assessed the effects of GtACR2 activation on hippocampal CA1 EPs. At the time and site of EP recording, GtACR2-mCherry was expressed in all layers of the CA1 subfield. Initially, before studying CA1 EPs, we characterized the light-induced potentials which were observed in the CA1 subfield following illumination in GtACR2 expressing rats. These potentials appeared as negative deflections both in the stratum pyramidale/oriens (axonal layer) and in the stratum radiatum (dendritic layer). Since they were not observed in Sham vector expressing animals, these potentials are a physiological phenomenon and not a light-artifact. The slope of these light-induced potentials increased with increasing LPD. As higher LPDs affect larger volumes of GtACR2 expressing tissue, the increased slope of the potentials most likely represents activation of more opsins. The negative slope of the extracellular field potentials is indicative for chloride efflux through activated GtACR2. We hypothesize that this chloride efflux is the result of isoflurane-induced hyperpolarization of the resting membrane potential (RMP) below the chloride equilibrium potential. Isoflurane activates TREK1 two pore domain K2P potassium channels which are abundantly expressed in hippocampal neurons and are important K^+^ leak channels that modulate the RMP to control excitability (Enyedi and Czirják, 2010; Comoglio et al., 2014; Pavel et al., 2020; Wague et al., 2020). Patch clamps studies indeed showed that isoflurane, at anesthetic levels, hyperpolarizes the RMP by several millivolts, while it does not affect the GABA_A_ reversal potential (Berg-Johnsen and Langmoen, 1990; Jones et al., 1992; Ou et al., 2020).

The initial slope of the light-induced potential was negative at both electrode contacts likely because GtACR2 was expressed in all compartments of pyramidal neurons and therefore its opening should result in the same potential change throughout the illuminated area of the CA1 subfield. The light-induced field potentials had a compound morphology indicating additional ionic currents such as efflux of potassium through activation of a type of voltage-gated potassium channel (K_v_) which are widely distributed in CA1 neurons (Storm, 1990). Various groups have reported that depolarization by activation of GtACR2 in neuronal compartments with reversed chloride gradients like axons could even generate action potentials (Mahn et al., 2016, 2018; Malyshev et al., 2017; Messier et al., 2018). However, in our experiment, no such observation could be confirmed.

An LPD-dependent increase in the inhibitory effect of GtACR2 activation was found on CA1 EPs, presumably due to increased light-distribution activating a larger volume of GtACR2 expressing CA1 neurons. The reduction in fEPSP slope and amplitude indicated a decrease in sodium influx into CA1 neurons. As well a larger effect was found on the neuronal firing by a reduction in the PS amplitude upon increasing LPDs. As in any optogenetic experiment, the magnitude of the effect is dependent on many factors such as properties of the opsin, expression levels, and LPD required to activate them *in vivo* (Yizhar et al., 2011). In our experimental conditions, an LPD of 160 mW/mm^2^ was sufficient for potent inhibition of EPs and beyond it, no added effect was observed. To compare GtACR2’s inhibitory efficiency *in vivo* with other opsins like eNpHR3.0, the spherical volume of inhibited tissue was calculated. Simulations indicated the extent of opsin-expressing tissue that could be modulated under identical illumination conditions is 67-fold higher for GtACR2 compared to eNpHR3.0. Hence, this increased sensitivity of GtACR2 is particularly useful in inhibiting a larger volume of neural tissue which could be seen from our experiment by suppression of PS, as well as it helps to prevent tissue heating by requiring lower light powers to activate the opsins *in vivo*.

Optogenetic inhibition of the first of two paired pulses given at an IPI of 20 ms shifted the paired-pulse relations from depression to facilitation. The mechanism responsible for PPD in a 20-ms paired-pulse EP protocol is due to GABA_A_-mediated feedback inhibition of CA1 pyramidal neurons (Leung et al., 2008). This feedback inhibition is resultant of the activation of inhibitory neurons innervating the CA1 neurons: SC stimulation activates CA1 neurons and CA1 axonal collaterals activate the interneurons. When the activation of CA1 neurons upon SC stimulation is optogenetically inhibited, this feedback inhibition is prevented. Besides the lack of feedback inhibition, PPF is most likely mediated by the increase in residual calcium levels at the active zones in the SC presynaptic terminals because of the first activation. The presence of residual calcium, from the first SC stimulation, causes more neurotransmitter to be released during a second SC stimulation (Regehr, 2012). This well-described form of short-term plasticity is not affected by the optogenetic inhibition of CA1 pyramidal neurons and should be prominent in the absence of feedback inhibition. Due to which a significant increase in P2 for all CA1 EP parameters during illumination was observed. These observations suggest precise activation of GtACR2 modulated the neuronal excitability by changing the excitation/inhibition balance of CA1 neuronal circuitry.

The inhibitory effects of GtACR2 briefly outlasted its activation. The effect was no longer present when the delay between the end of 10 ms light-pulse and the PS peak was 18 ms or longer. The ability of these chloride channel opsins for rapid and reversible suppression of postsynaptic neuronal firing makes them a useful tool for neuroscience research, where precisely timed interventions are necessary such as behavioral experiments, and for closed-loop systems where quick feedback is desired. Also, increasing the light-pulse durations sustained GtACR2’s inhibitory effect without enhancing it for any of the parameters of CA1 EPs. We hypothesize this to be the result of shunting inhibition by activation of GtACR2. When activated, optogenetic chloride channels like GtACR2 effectively clamp the neuronal membrane potential to the equilibrium potential of chloride and shunt membrane depolarization if chloride permeability is high (Mahn et al., 2018). This channel-mediated neuronal inhibition is different from chloride pump opsins that load neurons with chloride in a time-dependent process (Raimondo et al., 2012). Thereby, GtACR2 reduces non-physiological changes in the intracellular concentration of chloride ions while inhibiting neurons throughout the duration of illumination. These results affirm that ACRs like GtACR2 are valuable tools by offering an alternative approach to optogenetic inhibition of neuronal networks *in vivo*. Hence, researchers have directed their efforts to improve this class of opsins by discovering (RapACR) (Govorunova et al., 2018) and engineering red-shifted variants with even faster kinetics (FLASH) (Kato et al., 2018), and improving targeting to soma to increase the expression on cell surfaces and reduce paradoxical axonal excitability (stGtACR2) (Mahn et al., 2018; Messier et al., 2018).

## Data Availability Statement

The raw data supporting the conclusions of this article will be made available by the authors, without undue reservation.

## Ethics Statement

The animal study was reviewed and approved by the Ethical Committee on Animal Experiments of Ghent University (ECD: 16/31).

## Author Contributions

AA, LL, WV, WW, JD, KV, AM, PB, and RR contributed to the study design and analysis plan. AA obtained the data. AA, LL, and RR analyzed the data and prepared the manuscript. All authors reviewed the manuscript.

## Conflict of Interest

The authors declare that the research was conducted in the absence of any commercial or financial relationships that could be construed as a potential conflict of interest.
