# Supplementary material for: Attenuation of Hippocampal Evoked Potentials in vivo by Activation of GtACR2, an Optogenetic Chloride Channel
Source: Front Neurosci. 2021 Mar 29;15:653844. doi: 10.3389/fnins.2021.653844 (PMC8039138; doi:10.3389/fnins.2021.653844)
Supplement: Supplementary file 1 [file Data_Sheet_1.pdf]

Supplementary Material

Attenuation of hippocampal evoked potentials in-vivo by activation of GtACR2, an optogenetic chloride-channel

Anirudh R. Acharya, Lars Emil Larsen, Wouter Van Lysebettens, Wytse Jan Wadman, Jean Delbeke, Kristl Vonck, Alfred Meurs, Paul Boon, and Robrecht Raedt*

4BRAIN, Department of Head and Skin, Ghent University, Ghent, Belgium
* Corresponding Author: robrecht.raedt@UGent.be

Supplementary Method 1: Monte-Carlo simulation for light-distribution in rat brain:

The Monte-Carlo simulations were done using a freely available MATLAB script, OptogenSIM, provided by Liu et al. (2015). It utilized a standard MRI atlas of rat brain (voxel dimensions: 125 µm × 125 µm × 125 µm) with grey matter, white matter, and CSF-specific optical properties assigned to each individual voxel (Valdés-Hernández et al., 2011). Optical properties included absorption coefficient, μ_a[mm^{-1}]; scattering coefficient, μ_s[mm^{-1}]; anisotropy of scattering, g [dimensionless], to each individual voxel of a rat brain MRI. Reduced scattering coefficient was modelled as: $\mu'_s(\lambda)$ = (a*(λ/500 nm)-b, where the scaling factor 'a' equals the value of μ'_s at 500 nm wavelength and factor 'b' is the scattering power. Scattering coefficient was then derived as, µs = $\mu'_s/(1-g)$, where the 'g' is the anisotropy factor. The absorption co-efficient of grey and white matter is estimated as: $\mu_a(\lambda)$ = B*S*$\mu_a(HbO_2(\lambda))$ + B*(1-S)*$\mu_a(Hb(\lambda))$ + W*$\mu_a(H_2O(\lambda))$. Where B is mean blood content and S oxygen saturation, W is water content, and $\mu_a(HbO_2)$ and $\mu_a(HO_2)$ is absorption coefficient of water and blood, respectively. Various values for optical parameters used for simulations are provided in the table below, values derived for generic brain model were kept same as provided in script. The corresponding launch site of photons was identified on MRI atlas (AP: 5.0 mm, ML: 3.0 mm, DV: 2.0 mm relative to Bregma) and 10^6 photons were simulated for obtaining fluence contours. The results of the simulations were then overlayed on rat brain atlas (Paxinos & Watson, 1998) indicating approximate location of optic fiber, dorsal (black line) and ventral (grey line) contacts of bipolar electrode.

**Optical parameter**	**Value**		
Radius of optic fiber	0.1 mm		
Numerical aperture	0.39		
Light-power at optic fiber tip	1 mW (32 mW/mm^2), 5 mW (160 mW/mm^2), 10 mW (320 mW/mm^2)		
	**Gray matter**	**White matter**	**CSF**
Anisotropy factor, *g*	0.9	0.9	0.9
Mean blood content, *B*	0.028	0.028	0
Oxygen saturation, *S*	0.62	0.62	0
Water content, *W*	0.65	0.65	1.0
Scaling factor, *a*	2.37 mm^{-1}	5.05 mm^{-1}	0.24 mm^{-1}
Scattering power, *b*	1.15	0.7	1.0

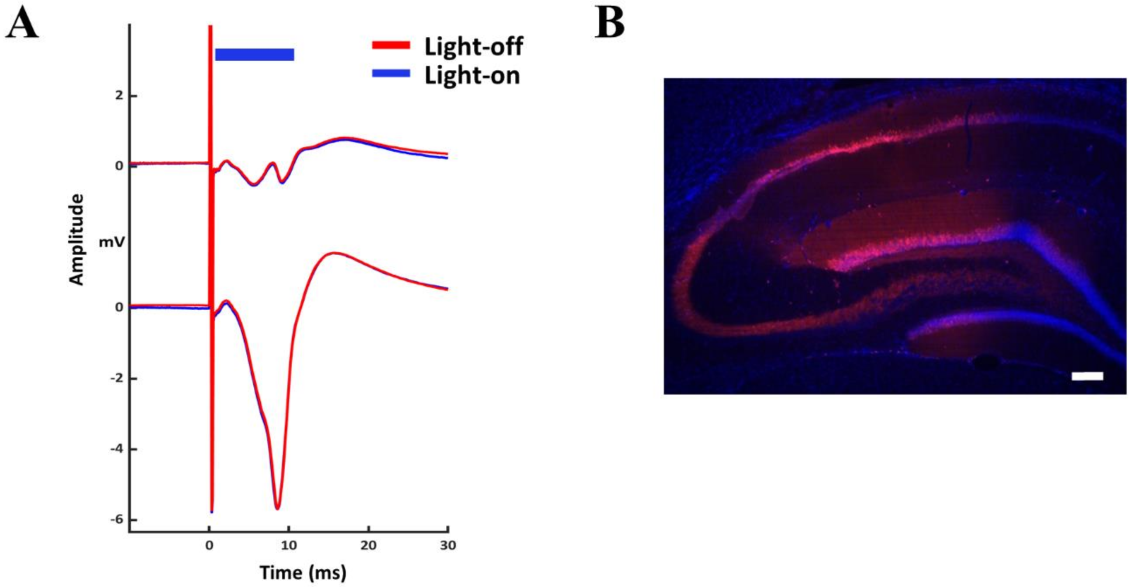

Supplementary Figure 1: No effect of illumination on EPs was found in the Sham group **(A)** Representative trace of EPs obtained in Sham group when illuminating CA1 subfield for 10 ms at LPD of 160 mW/mm^2. The dorsal contact measuring PS amplitude and ventral contact measuring fEPSP slope at light-off (red) and light-on (blue) conditions **(B)** Histological verification of mCherry expression (red) and nuclear stain DAPI (blue) scale bar measures 200 µm.

References:

Liu, Y., Jacques, S. L., Azimipour, M., Rogers, J. D., Pashaie, R., and Eliceiri, K. W. (2015). OptogenSIM: a 3D Monte Carlo simulation platform for light delivery design in optogenetics. *Biomed Opt Express* 6, 4859–4870. doi:10.1364/BOE.6.004859.

Paxinos, G., and Watson, C. (1998). *The Rat Brain in Stereotaxic Coordinates*. Elsevier.

Valdés-Hernández, P. A., Sumiyoshi, A., Nonaka, H., Haga, R., Aubert-Vásquez, E., Ogawa, T., et al. (2011). An in vivo MRI Template Set for Morphometry, Tissue Segmentation, and fMRI Localization in Rats. *Front Neuroinform* 5, 26. doi:10.3389/fninf.2011.00026.
